# Variation and Demographic Determinants of Malaria Infection in Selected Villages of Bibugn Woreda, Amhara, Ethiopia

**DOI:** 10.1155/japr/7884712

**Published:** 2026-09-06

**Authors:** Anmut Assemie

**Affiliations:** ^1^ Department of Biology, Wachemo University, Hossana, Ethiopia, wcu.edu.et

**Keywords:** Bibugn, demographic determinants, malaria prevalence, *Plasmodium* species

## Abstract

Malaria remains a major public health problem in Ethiopia, with heterogeneous transmission across ecological and demographic settings. This study assessed variation and demographic determinants of malaria infection in selected villages of Bibugn woreda, Amhara, Ethiopia. A community‐based cross‐sectional survey was conducted from October to December 2016 and March to April 2017 in Ameba Amarsa, Debersina, and Moseba Shemiabo villages, which were purposively selected from the woreda. A total of 422 residents were randomly selected from the purposively selected villages. Thick and thin blood smears were examined microscopically to detect *Plasmodium* species. Data were entered and analyzed by using SPSS software. Associations between malaria infection and predictor variables were analyzed using chi‐square tests at a 95% confidence level. Overall, 46 participants were positive, yielding a malaria prevalence of 10.9% (95% CI: 8.0%–14.3%). Infection was higher among males (67.39%) than females (32.61%), but not statistically significant (*χ* ^2^ = 1.753, df = 1, *p* = 0.185). The occurrence of *Plasmodium* species was not significant in residence (*χ* ^2^ = 3.609, df = 1, *p* = 0.058), but there is significant variation in age (*χ* ^2^ = 9.909, df = 2, *p* = 0.007). *Plasmodium vivax* was the predominant species, accounting for 60.87% (28/46) of infections, whereas *P. falciparum* comprised 39.13% (18/46). Debersina village exhibited the highest proportion of infections (69.56%), followed by Moseba Shemiabo (17.39%) and Ameba Amarsa (13.04%). Malaria transmission persists at a moderate level in Bibugn Woreda, with *P. vivax* emerging as the dominant parasite contrasting the national pattern of *P. falciparum* predominance. Strengthening surveillance, improving vector control coverage, and incorporating *P. vivax* specific strategies are critical for accelerating malaria elimination efforts in the district.

## 1. Introduction

Malaria remains one of the most important vector‐borne diseases worldwide and continues to constitute a major public health challenge, particularly in sub‐Saharan Africa. According to the World Health Organization (WHO), an estimated 263 million malaria cases and 597,000 deaths occurred globally in 2023, representing an increase of approximately 11 million cases compared with 2022. The WHO African Region accounted for 94% of global malaria cases and 95% of malaria deaths, highlighting the disproportionate burden borne by the continent [1]. Malaria is caused by five species of *Plasmodium*, of which *Plasmodium falciparum* and *Plasmodium vivax* are responsible for the majority of infections [1, 2].

Ethiopia remains among the malaria‐endemic countries in sub‐Saharan Africa. Approximately 75% of the country′s landmass is malarious, placing nearly 60 million people (about 50% of the population) at risk of malaria infection [3, 4]. Recent malaria stratification identified that 565 of the country′s 845 districts are malaria endemic, with transmission varying substantially across ecological and climatic zones [3, 4]. Although Ethiopia has achieved considerable progress through the scale‐up of insecticide‐treated nets, indoor residual spraying, rapid diagnostic testing, and artemisinin‐based combination therapy, malaria remains a major cause of outpatient visits and hospital admissions [1, 5].

Malaria transmission is influenced by a complex interaction of environmental, climatic, demographic, and socioeconomic factors. Temperature, rainfall, altitude, humidity, land use, population movement, housing conditions, and access to healthcare services affect vector abundance and parasite development, leading to considerable spatial and temporal heterogeneity in disease transmission [5–11]. Climate variability and environmental changes have also contributed to the expansion of malaria into areas previously considered unstable or low risk [8, 11].

In Ethiopia, *P. falciparum* and *P. vivax* account for approximately 60% and 40% of malaria cases, respectively [12–14]. However, substantial geographical variation in species composition has been reported in different parts of the country [15, 16]. The ability of *P. vivax* to form dormant liver stages and relapse contributes to its persistence and complicates malaria elimination efforts (38). Moreover, human mobility and occupational exposure can alter local transmission dynamics and parasite distribution [5].

Demographic characteristics also play an important role in malaria epidemiology. Age, sex, residence, occupation, migration, and socioeconomic conditions have been recognized as important determinants of malaria risk [5, 7, 17]. In stable transmission settings, children under 5 years and pregnant women generally experience the greatest disease burden, whereas adults engaged in agricultural and outdoor activities may experience increased exposure in unstable transmission areas [18].

The Ethiopian government aims to eliminate malaria by 2030, with the National Malaria Elimination Roadmap (2021–2025) targeting interruption of transmission in low‐transmission districts and strengthening surveillance systems [4].

Therefore, the present study is aimed at assessing malaria prevalence, *Plasmodium* species composition, and selected demographic characteristics associated with malaria infection among residents of three villages in Bibugn Woreda, Amhara Region, Ethiopia. The findings provide baseline epidemiological information and contribute to understanding local variation in malaria transmission, thereby supporting future surveillance and malaria elimination efforts in Ethiopia [4].

## 2. Materials and Methods

### 2.1. Description of Study Area

The study was conducted in three villages of Bibugn Woreda, East Gojjam Zone, Amhara Region, Ethiopia: Ameba Amarsa (10°58 ^′^28 ^″^ N, 37°46 ^′^42 ^″^ E), Debersina (11°00 ^′^19 ^″^ N, 37°45 ^′^23 ^″^ E), and Moseba Shemiabo (10°59 ^′^04 ^″^ N, 37°43 ^′^56 ^″^ E). The area lies at an altitude of 1250–2500 m above sea level and comprises predominantly Kola and Weinadega agroecological zones. The district has a predominantly rural population, mainly of Amhara ethnicity and Ethiopian Orthodox faith. Mixed crop‐livestock farming is the principal livelihood activity. Warm temperatures, seasonal rainfall, and the presence of temporary water bodies provide favorable conditions for mosquito breeding and malaria transmission [19, 20].

### 2.2. Study Design

The study sites were purposively selected based on altitude and temperature, with elevations ranging from 1250 to 2500 m above sea level and temperatures between 21°C and 29°C. A community‐based cross‐sectional study was conducted to assess malaria infections in the selected villages. The sample was collected from October to December 2016 and March to April 2017 from Ameba Amarsa, Debersina, and Moseba Shemiabo villages.

### 2.3. Study Participants

For blood sample collection, 422 study participants who visit the health centers, from the purposively selected villages for any kind of health service during the study period, were selected randomly as the study population. Those who permanently lived in the study area were included as study participants, but individuals with the clinical symptom of malaria and those taking antimalarial drugs during data collection were excluded.

### 2.4. Blood Sample Collection and Identification

Parasitological examination was conducted by experienced laboratory technicians using capillary blood samples collected via finger‐prick from study participants. Blood smears were prepared according to the WHO standard protocol. All prepared slides were securely transported to the district malaria control laboratory, where microscopic examination was performed according to WHO guidelines [21]. Parasite density was determined from thick blood smears, whereas species identification was performed using thin smears [22]. Thick films were examined under high‐power magnification to detect the presence of malaria parasites, and thin films were observed under a 1000× oil immersion objective for species identification. A minimum of 100 consecutive fields on each thick film were examined before declaring a slide negative [23].

### 2.5. Sample Size Determination

The sample size was calculated using a single population proportion formula at a 95% confidence level (Z*α*/2 = 1.96). In the absence of prior studies in the area and because clinic data were only available after the epidemiological study, a prevalence (P) of 50% was assumed. Using a 5% margin of error (d), the minimum required sample size was estimated to be 384 participants, as shown below.

n=Z22 P 1−P/d

n=Z222ά/50%50%/d

n=1.961.960.50.5/0.05

n=384,

where *n* is the sample size; *P* is the average prevalence; Zά/2 is the *p* value at 95% CI from table; and *d* is the marginal error.

To account for potential nonresponse, a 10% contingency was added to the minimum sample size, resulting in a total of 422 study participants being enrolled.

### 2.6. Data Analysis

Data from blood film examinations and associated parasitological findings were entered and analyzed using SPSS statistical software. Differences in malaria prevalence between urban and rural villages, as well as across sociodemographic variables, were assessed using the chi‐square test. A *p* value of less than 0.05 was considered statistically significant.

## 3. Results

### 3.1. Sociodemographic Profile of Participants

In this study, a total of 422 individuals participated, including 246 males and 176 females. The majority of participants (75.6%) were residents of rural villages as presented in Table 1.

**Table 1:** Sociodemographic profile of participants.

Variables	Participant profile	%
Sex		
Male	246	58.29%
Female	176	41.71%
Residence		
Rural	319	75.59%
Urban	103	24.41%
Age		
< 5	76	18%
5–14	134	31.75%
≥ 15	212	50.24%
Total	422	100%

### 3.2. Malaria Infection Rates

Out of 422 individuals screened, 46 (10.9%) showed evidence of malaria infection. Among the malaria‐positive cases*, P. vivax* predominated (60.87%) over *P. falciparum* (39.13%). Malaria infection was higher in males (31/246; 12.6%) than in females (15/176; 8.5%), but the difference was not statistically significant (*χ* ^2^ = 1.753, df = 1, *p* = 0.185). Rural residents had a higher prevalence (40/319; 12.5%) than urban residents (6/103; 5.8%), although the observed difference was not statistically significant (*χ* ^2^ = 3.609, df = 1, *p* = 0.058). Malaria prevalence differed significantly across age groups (*χ* ^2^ = 9.909, df = 2, *p* = 0.007), with the highest prevalence observed among individuals aged ≥ 15 years (33/212; 15.6%), followed by the < 5 years (6/76; 7.9%) and 5–14 years (7/134; 5.2%) age groups. Across all groups, *P. vivax* was the dominant species (Table 2).

**Table 2:** Association of malaria infection with sex, residence, and age group.

Variables	Number screened	Malaria case		
		Positive	*X* ^2^	*p*
Sex				
Male	246	31		
Female	176	15	1.753	0.185
Residence				
Rural	319	40	3.609	0.058
Urban	103	6		
Age				
< 5	76	6		
5–14	134	7	9.909	0.007
≥ 15	212	33		
Total	422	46		

### 3.3. *Plasmodium* Species Profile

Out of 422 participants, 46 (10.9%) were confirmed to have malaria. *P. vivax* was the most frequently detected species, comprising 60.87% of infections, whereas *P. falciparum* accounted for 39.13%. Males accounted for 67.39% of cases, whereas females accounted for 32.61%, with no significant difference between sexes (*χ* ^2^ = 0.007, df = 1, *p* = 0.933). The majority of infections occurred in rural residents (86.95%) compared with urban residents (13.04%), with no significant difference by residence (*χ* ^2^ = 0.097, df = 1, *p* = 0.755). The highest malaria prevalence was observed in participants aged ≥ 15 years (71.74%), with the next highest in the 5–14 years age group (15.21%) and < 5 years (13.04%), with a statistically significant difference across age groups (*χ* ^2^ = 6.924, df = 2, *p* = 0.031). Across all groups, *P. vivax* was the dominant species (Table 3).

**Table 3:** The *Plasmodium* species profile during the study periods.

Variables	Total screened	*Plasmodium* species			*X* ^2^	*p*
		*P. falciparum*	*P. vivax*	Total		
Sex						
Male	246	12 (26.09%)	19 (41.30%)	31 (67.39%)		
Female	176	6 (13.04%)	9 (19.57%)	15 (32.61%)	0.007	0.933
Residence						
Rural	319	16 (34.78%)	24 (52.17%)	40 (86.95%)		
Urban	103	2 (4.35%)	4 (8.7%)	6 (13.04%)	0.097	0.755
Age						
< 5	76	4 (8.7%)	2 (4.35%)	6 (13.04%)		
5–14	134	5 (10.86%)	2 (4.35%)	7 (15.21%)	6.924	0.031
≥ 15	212	9 (19.57%)	24 (52.17%)	33 (71.74%)		
	422	18 (39.13%)	28 (60.57%)	46 (100%)		

### 3.4. Distribution of Malaria Parasite Species

Among the total *Plasmodium* cases, 13.04% were recorded in Ameba Amarsa village, 69.56% in Debersina village, and 17.39% in Moseba Shemiabo village. Of the infected males (67.39% of total cases), 8.7% were from Ameba Amarsa, 47.82% from Debersina, and 10.87% from Moseba Shemiabo. Among infected females, 4.35% were from Ameba Amarsa, 21.74% from Debersina, and 6.52% from Moseba Shemiabo. Debersina village showed the highest prevalence of *Plasmodium* infections among the study sites (Table 4). In Ameba Amarsa, infection rates were 6.5% in males and 4.2% in females (*χ* ^2^ = 0.010, df = 1, *p* = 0.920), whereas in Debersina, 16.4% of males and 12.2% of females were infected (*χ* ^2^ = 0.423, df = 1, *p* = 0.515); in both villages, the gender differences were not statistically significant. Likewise, in Moseba Shemiabo, infection prevalence was 10.0% among males and 6.5% among females, showing no significant difference (*χ* ^2^ = 0.061, df = 1, *p* = 0.805).

**Table 4:** Distribution of malaria infection by sex across study area.

Villages	Sex	No. examined	No. positive	No. negative	% positive	*χ* ^2^	*p*
Ameba Amarsa	Male	62	4	58	6.5%		
	Female	48	2	46	4.2%	0.010	0.920
Subtotal		110	6	104	5.5%		
Debersina	Male	134	22	112	16.4%		
	Female	82	10	72	12.2%	0.423	0.515
Subtotal		216	32	184	14.8%		
Moseba Shemiabo	Male	50	5	45	10.0%		
	Female	46	3	43	6.5%	0.061	0.805
Subtotal		96	8	88	8.3%		
Grand total		422	46	376	10.9%		

## 4. Discussion

The present research was conducted to determine malaria prevalence, *Plasmodium* species distribution, and associated demographic factors in the selected villages. Among the 46 malaria‐positive cases identified in this study, 60.87% were *P. vivax* and 39.13% were *P. falciparum*. Although *P. falciparum* remains the predominant malaria parasite nationally, the predominance of *P. vivax* observed in the present study indicates local heterogeneity in parasite distribution [1, 12–14]. Similar findings have been reported from Southern Ethiopia and other parts of the country, where *P. vivax* constituted a substantial proportion of infections [14, 24, 25]. Differences from the national pattern may reflect ecological variations, seasonal fluctuations, population characteristics, and differences in diagnostic methods [8, 11].

The results of this study show partial agreement with earlier reports from Southern Ethiopia documented in the Health and Health‐Related Indicators (1992). That report identified 61,079 confirmed malaria cases, of which 47.2% were *P. falciparum* and 50.9% were *P. vivax*, illustrating the relatively lower occurrence of *P. falciparum* in the area. Likewise, a study from Assendabo Health Center found that 67.5% of malaria cases were due to *P. vivax*, whereas 32.5% were attributed to *P. falciparum*, further supporting the patterns observed in the present study [26].

This study aligns with earlier findings from Akaki (1995), where 2136 samples were examined and 78 (5.7%) were positive for malaria. Among these, 54 cases (69%) were attributed to *P. vivax* and 24 cases (31%) to *P. falciparum* [27]. Similarly, comparable results were reported in a 2007 study conducted in Aleta Wondo involving 185 participants, in which 103 individuals (55.7%) were malaria‐positive; of these, 68 (66%) were infected with *P. vivax* and 35 (34%) with *P. falciparum* [24]. Consistent findings were also documented at Hallaba Health Center in Southern Ethiopia in 2014, where 169 of 204 participants (82.84%) were positive for *Plasmodium* infection, with *P. vivax* accounting for 119 cases (70.41%) and *P. falciparum* for 39 cases (23.08%) [25].

The relatively higher proportion of *P. vivax* infections in these studies may be linked to its biological capacity to form dormant liver stages (hypnozoites), which can lead to relapse and sustained transmission even when overall malaria incidence declines [28]. Moreover, human mobility and occupational exposure are likely to contribute to the continued circulation and introduction of infections in affected communities [5, 7, 11].

Contrary to the typical age distribution observed in highly endemic settings, where children under 5 years of age are disproportionately affected [18], the present study found significantly higher malaria prevalence among individuals aged 15 years and above. This deviation from the conventional pattern may be related to increased occupational and behavioral exposure among adults. Individuals in this age category are more likely to participate in agricultural activities and outdoor nighttime work, increasing their contact with *Anopheles* mosquitoes. Population mobility and travel to endemic areas may also contribute to the increased prevalence among adults [7, 9].

Malaria prevalence was higher among males than females, although the difference was not statistically significant. Similar observations have been reported from East Gojjam and other parts of Ethiopia [16, 17]. The slightly higher prevalence among males may be attributable to greater participation in outdoor evening and nighttime activities, which increase exposure to mosquito bites [7]. However, the absence of a significant association suggests that sex alone was not an important determinant of malaria infection in the study population.

Likewise, malaria prevalence was higher among rural residents than urban residents, although the difference did not reach statistical significance. This finding may be explained by differences in housing conditions, vector breeding habitats, agricultural practices, and accessibility of malaria control interventions [6–9]. Rural communities are often located closer to mosquito breeding sites and may experience greater exposure to infective vectors.

Marked variation in malaria prevalence was observed among the three villages, with Debersina exhibiting the highest proportion of infections. Such variation suggests spatial heterogeneity in malaria transmission within the district. Differences in altitude, local climatic conditions, land use patterns, and proximity to vector breeding habitats may partly explain these variations [8, 29]. Because household geographic coordinates and detailed environmental information were unavailable during the study period, advanced spatial analyses such as Geographic Information System (GIS)–based mapping and hotspot analysis could not be performed. Future studies integrating geospatial tools and environmental variables would provide a more comprehensive understanding of malaria transmission dynamics in the area.

The overall prevalence of malaria in the study area was 10.9%, which is notably higher than previous reports from Oromia and the Southern Nations, Nationalities, and Peoples′ Regions (2.4%) [30], the Amhara Regional State (4.6%) [31], Woyn Wuha Health Center in East Gojjam, Amhara Regional State [32], and South West Ethiopia (10.5%) [16]. Such differences may arise from variations in environmental conditions, malaria transmission intensity, population structure, diagnostic approaches, and study periods [8, 11, 23]. Since malaria transmission in Ethiopia is characterized by substantial temporal and spatial heterogeneity, direct comparisons among studies should be interpreted with caution.

Seasonal variation may also have contributed to the observed prevalence pattern. Data collection was conducted during October–December 2016 and March–April 2017, corresponding to the major and minor malaria transmission seasons in Ethiopia. Malaria transmission was particularly elevated during October and November, following the main rainy season, when increased rainfall creates favorable breeding conditions for *Anopheles* mosquitoes [6, 8, 23]. This seasonal pattern is consistent with previous reports from Ethiopia showing increased malaria transmission after periods of heavy rainfall [23, 33].

Only two *Plasmodium* species, *P. vivax* and *P. falciparum*, were detected during the study period, despite the occurrence of additional human malaria species in Ethiopia [30, 34]. The absence of other species may reflect their low prevalence in the area or temporal fluctuations in species distribution. Standard microscopic techniques based on WHO protocols were used for diagnosis and remain the reference method for malaria detection [21, 22]. Nevertheless, more advanced approaches such as molecular techniques and geospatial analyses could provide additional insights into species diversity and transmission patterns.

These findings underscore the importance of considering local epidemiological heterogeneity when implementing interventions aimed at achieving the Ethiopian malaria elimination targets outlined in the National Malaria Elimination Roadmap 2021–2025 [4].

Although the present study assessed variation in malaria prevalence among geographically distinct villages, advanced spatial analytical techniques such as GIS‐based mapping and spatial cluster analysis were not performed because household‐level geographic coordinates and detailed environmental spatial data were not available during the study period. Nevertheless, the observed variation in malaria prevalence among the study villages suggests possible spatial heterogeneity in malaria transmission. Future studies integrating GIS technology, spatial autocorrelation, hotspot analysis, and environmental risk factors would provide a more comprehensive understanding of malaria distribution and transmission dynamics in the study area.

## 5. Conclusion

This study provides a baseline assessment of malaria infection in the study area across the three selected villages. The findings showed that malaria cases were attributed solely to two *Plasmodium* species, *P. falciparum* and *P. vivax*, with *P. vivax* being the more prevalent species. The highest infection burden was recorded among individuals aged 15 years and above. Overall, these results suggest that the distribution of *P. vivax* and *P. falciparum* in the study area differs from the national pattern.

In addition, chi‐square analysis conducted for each village as well as the combined dataset indicated no statistically significant difference in malaria infection between males and females. This implies that sex was not a determining factor for malaria infection risk in the study area during the study period.

## Author Contributions

The author was responsible for data collection, data management, parasite identification, and the assessment of malaria prevalence. Except for blood sample collection, staining procedures, and external technical comments, all components of the study were carried out by the author.

## Funding

No funding was received for this manuscript.

## Ethics Statement

Ethical clearance for the study was obtained from the university. Permission to collect blood samples from participants was granted by the director of the district health center. Individuals who tested positive for malaria received appropriate treatment: Coartem for *Plasmodium falciparum* infections and chloroquine for *Plasmodium vivax* infections. Prior to participation, the purpose and significance of the study, as well as potential risks and benefits, were clearly explained to all participants. Written and verbal informed consent was obtained from each volunteer before sample collection. For participants under 18 years of age, written consent was obtained from their parents or guardians. Only individuals who voluntarily agreed to participate were included in the study.

## Conflicts of Interest

The author declares no conflicts of interest.

## Data Availability

All supporting data for the findings reported in this study are included in the manuscript.
